# Reply to Bajpai et al., “Selective digestive decontamination and antimicrobial resistance: defining thresholds before global implementation”

**DOI:** 10.1128/aac.00712-26

**Published:** 2026-07-21

**Authors:** Wanda Cornistein, Maria Inés Staneloni, on behalf of the PREVAR Study Group

**Affiliations:** 1 Hospital Universitario Austral 218809, Buenos Aires, Argentina; 2 Hospital Italiano de Buenos Aires 37533, Buenos Aires, Argentina; Houston Methodist Hospital and Weill Cornell Medical College, Houston, Texas, USA

## REPLY

We sincerely thank you for your thoughtful letter (1) and for the interest you have shown in our study. This kind of scholarly exchange is precisely what advances our collective understanding of such a complex and pressing issue, and we are truly grateful for it.

While selective digestive decontamination (SDD) was not among the practices evaluated in our prevalence study, we feel it is important to underscore that SDD is not recommended in Argentina, and the data from our study help explain why. Argentina is a setting where polymyxins and aminoglycosides remain clinically relevant agents—not only as last-resort therapies for serious infections caused by multidrug-resistant organisms (MDROs), sometimes used in combination regimens when no newer alternatives are available, but also for the management of less severe MDRO infections. In this context, the prophylactic use of these agents as part of an SDD regimen would represent a risk of accelerating resistance to drugs that still carry critical therapeutic value in our region.

We fully agree with your call for a consensus-based quantitative definition of “low antimicrobial resistance prevalence” to guide SDD eligibility. As currently formulated, recommendations leave considerable room for subjective interpretation, which could lead to inappropriate implementation in settings where SDD may cause more harm than benefit. In this regard, we believe our study offers a meaningful reference point: the finding that approximately 25% of patients admitted to Argentine ICUs are colonized by MDROs provides a concrete, measurable benchmark that could contribute to building such a framework.

Regarding your proposal to conduct dedicated randomized controlled trials assessing SDD in LMIC ICUs with high MDRO prevalence, given the ecological conditions we have described—high baseline resistance rates, limited access to new drugs, and the ongoing clinical reliance on polymyxins and aminoglycosides—we are concerned that such trials could carry significant risks in terms of further resistance selection. The potential consequences in settings already under considerable therapeutic pressure could be severe, and this must be carefully weighed before any such trials are designed or initiated.

Finally, we would like to highlight that, even in high-resistance settings, such as ours, meaningful reductions in ventilator-associated pneumonia (VAP) rates are achievable without resorting to SDD. Our group has previously demonstrated this through the implementation of an experiential learning-based quality improvement strategy (2), reinforcing that non-pharmacological, bundle-based approaches can be both effective and safe in resource-constrained, high-resistance environments.

Once again, we thank you for this exchange.
